# Cumulative exposure to tacrolimus during early period after liver transplantation does not affect the recurrence of hepatocellular carcinoma

**DOI:** 10.1038/s41598-023-46803-8

**Published:** 2023-11-19

**Authors:** Deok-Gie Kim, Seung Hyuk Yim, Eun-Ki Min, Mun Chae Choi, Dong Jin Joo, Myoung Soo Kim, Jae Geun Lee

**Affiliations:** Department of Surgery, The Research Institute for Transplantation, Yonsei University College of Medicine, 50-1 Yonsei-ro, Seodaemun-gu, Seoul, 03722 South Korea

**Keywords:** Gastrointestinal cancer, Transplant immunology

## Abstract

The clinical effects of tacrolimus (TAC) exposure on hepatocellular carcinoma (HCC) recurrence after liver transplantation (LT) remain unclear. In this retrospective single centric study, 512 patients who underwent LT for HCC were divided into four groups according to cumulative exposure to tacrolimus (CET) during 3 months after LT: conventional (n = 218), aggressive minimization (n = 32), minimization (n = 161), and high exposure (n = 101). Impact of CET on HCC recurrence and death were analyzed. Compared with the conventional group, the other three CET groups showed a similar risk of HCC recurrence. The aggressive minimization group showed a higher risk [hazard ratio (HR) 5.64, P < 0.001] and the high exposure group showed a marginal risk (HR 1.67, P = 0.081) of overall death compared to the conventional group. CET during 3 months was not associated with HCC recurrence in the matched cohort and various subgroups. TAC minimization is not effective to prevent HCC recurrence but could result in higher mortality in LT recipients.

## Introduction

Liver transplantation (LT) is an optimal treatment option for unresectable hepatocellular carcinoma (HCC), with adequate patient selection^1^. However, the overall recurrence of HCC is reported to be up to 15% after LT; therefore, reducing the recurrence of HCC has been an important concern in LT patients^2^. While the importance of pre-transplant locoregional treatment and selection criteria for LT have been broadly investigated^3–7^, there is still a considerable knowledge gap regarding the role of post-transplant management in the recurrence of HCC^8^.

Tacrolimus (TAC) is the mainstay of immunosuppression for preventing rejection in LT recipients^9^. TAC was revealed to have a pro-oncogenic effect in an in vitro model^10,11^; therefore, research has focused on minimizing TAC exposure to reduce HCC recurrence. A retrospective study reported lower HCC recurrence in patients with low exposure to TAC compared to those with high exposure^12^. However, a subsequent multicenter randomized trial (SiLVER) did not show improved HCC recurrence-free survival, except in the low-risk population^13^. Another randomized trial comprising approximately 20% of LT recipients with HCC (H2304 and H2307) has not yet reached robust results regarding HCC-related outcomes^14^. Furthermore, these studies focused on mTOR inhibitors (mTORi) rather than TAC exposure itself. Therefore, the clinical effect of TAC concentration on HCC recurrence after LT remains unclear.

Because TAC is known to have intra-patient variability^15^, exact exposure to TAC should be evaluated based on the integrated parameter of every serum trough level rather than a single measurement at a specific time point or mean/median value. Rodriguez-Peralvarez et al. introduced a novel approach of cumulative exposure to TAC (CET) to demonstrate the effect of TAC exposure on renal function and de novo malignancy^16,17^. However, CET has not yet been widely validated for HCC outcomes after LT. Thus, this study aimed to analyze the effects of CET during early period after LT on HCC recurrence and survival.

## Materials and methods

### Study population and data collection

**Figure 1:**
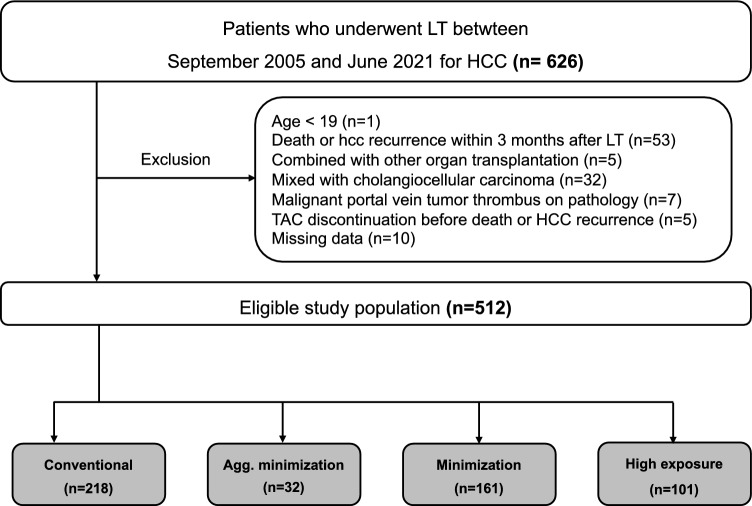
Study population. (**a**) CCC (n = 13), mixed HCC and CCC (n = 56), metastatic cancer(n = 2), hepatoblastoma (n = 2), and sarcomatoid HCC (n = 2). *CCC* cholangiocellular carcinoma, *HCC* hepatocellular carcinoma.

This retrospective single-center observational study was performed using data from patients who underwent LT for HCC between January 2006 and December 2021. Patients were followed up until death, loss to follow-up, 60 months after LT, or Jun 30^th^, 2022 (whichever came first). From a total of 626 subjects, the exclusion criteria were as follows: age < 18 years (n = 1), death or HCC recurrence within 90 days (n = 53), combined solid organ transplantation (n = 5), mixed cholangiocellular carcinoma (n = 32), malignant portal vein tumor thrombus on pathology (n = 7), TAC discontinuation before death or HCC recurrence (n = 5), and missing data (n = 10). Finally, 512 LT recipients were analyzed (Fig. 1). Recipient and donor baseline characteristics as well as tumor pathology were prospectively collected from our institutional database. TAC trough levels and the use of each immunosuppressant were extracted from our electronic medical records.

### Immunosuppression protocol

According to our institutional policy, TAC was started between -1 and 1 post-operative day with minimal exceptions. The serum trough level of TAC was assessed everyday for the first week and then 2–3 times a week until discharge. Thereafter, the TAC level was checked at every outpatient visit at an interval of 1–2 weeks for 2–3 months then every month, and finally every 3 months as follow-up time went on. Induction treatment was performed using an interleukin-2 receptor inhibitor. Steroids were initiated at a dose of 500 mg or 1000 mg on the LT date and then tapered to 5–10 mg around 14 postoperative days. Mycophenolate mofetil (MMF) was optionally added to the regimen according to the risk of rejection or infection, possibly starting on postoperative day 2. An mTOR inhibitor was used as an alternative to MMF after at least 21 postoperative days, according to our institutional indications: 1) higher risk of HCC recurrence, 2) suspected chronic renal injury, and 3) adverse effects of MMF in patients who needed additional maintenance other than TAC and steroids.

### Tacrolimus exposure

**Figure 2:**
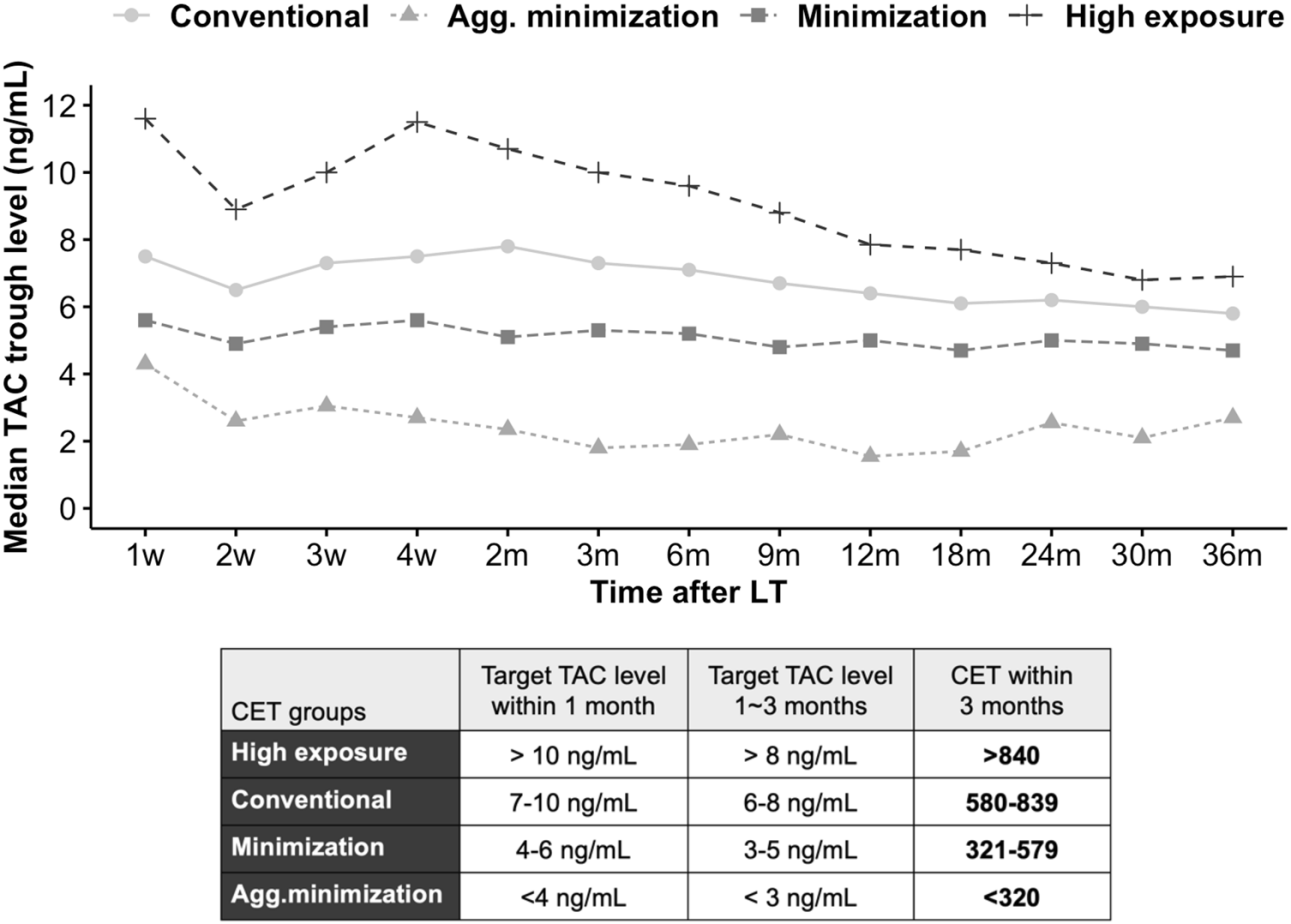
Tacrolimus trough levels in 4 CET groups. *Agg.minimization* aggressive minimization, *CET* cumulative exposure to tacrolimus, *TAC* tacrolimus.

We calculated CET according to the area under the curve, which was delineated from all measured trough levels of TAC during the first 3 months after LT in each patient, using the method previously reported by Rodriguez-Peralvarez et al.^16^. Patients were categorized into four groups by CET within 3 months, according to the target trough levels during the period: aggressive minimization (aggregate minimization, CET < 320), minimization (CET 321–579), conventional (CET 580–839), and high exposure (CET > 840). Equivalent TAC trough levels for each CET group were presented in Fig. 2. Other parameters indicating intra-patient variability of TAC within 3 months were calculated, such as the standard deviation, variance, and coefficient of variation (standard deviation/mean)^15^.

### Outcomes

The primary outcome was HCC recurrence, which was recorded when HCC was first identified by imaging studies. Patients were screened by computed tomography, ultrasonography, or magnetic resonance imaging along with alpha-feto protein (AFP) and protein induced by vitamin K absence or antagonist-II (PIVKA II) at least every 3 months for the first year after LT, at 3–6 months intervals until 2 years, and then annually. The secondary outcomes were overall mortality and acute rejection.

### Statistical methods

Categorical variables were presented as numbers (proportions) and compared between groups using the chi-square test. Continuous variables were presented as a median (interquartile range [IQR]) and compared using the Kruskal–Wallis test. HCC recurrence-free survival and overall survival were compared between the groups using the Kaplan–Meier analysis with the log-rank test. Multivariable Cox regression was performed, including the CET group and other covariates with P values < 0.10 in univariable analyses in the model. When analyzing HCC recurrence, death from non-HCC causes was treated as a competing risk according to the Fine and Gray method^18^. To further confirm the effect of the CET group on HCC recurrence, matched analyses were performed between the conventional group and the other three CET groups, the detailed method of which is described in the supplementary material.

The effect of CET on HCC recurrence was analyzed in various subgroups using Kaplan–Meier analyses only in univariable settings because of the small number of events in each subgroup. Risk factors for acute rejection during the first year after LT were evaluated using multivariable logistic regression, including covariates selected in a stepwise fashion. All analyses were performed using the R statistical package, version 4.2.0, for macOS (http://cran.r-project.org), with the threshold for significance set at *P* < 0.05.

### Ethical approval

This study was performed following the Declaration of Helsinki and the Declaration of Istanbul. Study design was approved and the requirement for informed consent from the study subjects was waived by the Institutional Review Board at Severance Hospital, Yonsei University Health System (IRB No. 4-2022-3156).

## Results

### Baseline characteristics

The baseline characteristics of the four groups are shown in Table 1. The aggressive minimization and minimization groups were older than the other two groups. The most common underlying liver disease for HCC was hepatitis B in all groups, with significant differences. Pretransplant model for end-stage liver disease (MELD) scores were significantly different between the four groups with a minimal gap in median values. All groups showed a living-donor predominance of > 75%. Donor age, sex, and graft steatosis were similar between groups. The median AFP for the conventional, aggressive minimization, minimization, and high exposure groups were 6.5, 8.0, 5.6, and 11.0 ng/mL, respectively (P = 0.009). The PIVKA II at LT were 31.5, 113.5, 38.0, and 28.0 mAU/mL, respectively (P < 0.001) and were significantly different between the groups, with higher values in the aggressive minimization group. The type of bridging therapy was significantly different between groups, with a higher incidence of systemic or radiotherapy in the aggressive minimization and minimization groups (11.9%, 37.5%, 17.4%, and 5.9%, respectively; P < 0.001). Regarding explant pathology, most variables were similar between groups, except for poor differentiation and satellite nodules. The four groups had similar HCC risk categories according to the Milan criteria and the French risk score.

**Table 1:** Baseline characteristics.

Variables	Conventional (n = 218)	Agg.minimization (n = 32)	Minimization (n = 161)	High exposure (n = 101)	P
Age, year	55.5 (51.0–61.0)	57.5 (51.0–64.5)	57.0 (53.0–63.0)	53.0 (49.0–59.0)	< 0.001
Sex, female	39 (17.9)	9 (28.1)	34 (21.1)	14 (13.9)	0.277
BMI, kg/m^2^	23.9 (22.3–25.8)	23.7 (21.6–26.1)	24.0 (22.1–26.5)	24.2 (22.5–25.8)	0.797
Underlying for HCC					0.029
Hepatitis B	168 (77.1)	22 (68.8)	117 (72.7)	91 (90.1)	
Hepatitis C	21 (9.6)	3 (9.4)	14 (8.7)	4 (4.0)	
Non-B, Non C	29 (13.3)	7 (21.9)	30 (18.6)	6 (5.9)	
ABO incompatibility	41 (18.8)	5 (15.6)	28 (17.4)	6 (5.9)	0.026
Hypertension	58 (26.6)	16 (50.0)	56 (34.8)	22 (21.8)	0.004
Diabetes mellitus	54 (24.8)	9 (28.1)	41 (25.5)	21 (20.8)	0.807
Pre-transplant MELD	10 (8–14)	11 (8–20)	12 (8–16)	9.0 (7–12)	0.001
Donor type					0.284
Living	170 (78.0)	26 (81.2)	135 (83.9)	75 (74.3)	
Deceased	48 (22.0)	6 (18.8)	26 (16.1)	26 (25.7)	
Donor age, year	32.0 (25.0–43.0)	42.0 (30.5–50.5)	34.0 (25.0–43.0)	33.0 (25.0–43.0)	0.107
Donor sex, female	81 (37.2)	14 (43.8)	66 (41.0)	30 (29.7)	0.225
Graft steatosis > 10%	5.0 (0.0–5.0)	5.0 (0.0–5.0)	5.0 (0.0–5.0)	5.0 (0.0–5.0)	0.689
AFP, ng/mL	6.5 (3.5–21.8)	8.0 (3.3–28.9)	5.6 (2.9–18.7)	11.0 (4.0–52.7)	0.009
PIVKA II, mAU/mL	31.5 (18.0–81.0)	113.5 (34.0–320.0)	38.0 (22.0–95.0)	28.0 (17.0–65.0)	< 0.001
Salvage LT	36 (16.5)	2 (6.2)	17 (10.6)	19 (18.8)	0.104
Bridging treatment					< 0.001
None	116 (53.2)	12 (37.5)	94 (58.4)	73 (72.3)	
Locoregional	76 (34.9)	8 (25.0)	39 (24.2)	22 (21.8)	
Systemic or radiotherapy	26 (11.9)	12 (37.5)	28 (17.4)	6 (5.9)	
Total necrosis	37 (17.0)	5 (15.6)	33 (20.5)	13 (12.9)	0.454
Viable tumor number	1.0 (1.0–3.0)	1.0 (1.0–4.0)	1.0 (1.0–3.0)	2.0 (1.0–3.0)	0.454
Maximum viable tumor size	1.7 (1.0–2.5)	1.8 (1.1–3.5)	1.6 (0.9–2.6)	1.9 (1.0–3.0)	0.361
Sum of viable tumor size	2.5 (1.0–4.5)	2.2 (1.1–6.5)	2.3 (0.9–5.1)	3.0 (1.3–5.7)	0.348
Microvascular invasion	44 (20.2)	8 (25.0)	35 (21.7)	27 (26.7)	0.533
Poor differentiation	53 (24.3)	16 (50.0)	58 (36.0)	26 (25.7)	0.003
Satellite nodule	16 (7.3)	3 (9.4)	14 (8.7)	18 (17.8)	0.030
Above Milan criteria	58 (26.6)	11 (34.4)	44 (27.3)	29 (28.7)	0.702
French risk score > 2	52 (23.9)	12 (37.5)	42 (26.1)	33 (32.7)	0.205

*AFP* alpha-feto protein, *HCC* hepatocellular carcinoma, *MELD* model for end-stage liver disease, *PIVKA II* protein induced by vitamin K absence or antagonist-II.

### Immunosuppressants

Among the entire study population, TAC was initiated before at least 4 postoperative days. Interleukin-2 receptor antibody was used for induction therapy in almost all patients except for one, with a total dose of 40 mg in 99.0% of the recipients. Three months after LT, 33.3% of patients were on MMF, while 31.8% were on mTOR inhibitors (Fig. S1).

The median tacrolimus trough level was well-stratified by CET group throughout the study period (Fig. 2). Parameters related to intra-patient variability showed significant differences among groups. Particularly, a higher coefficient of variation was observed in the aggressive minimization group than those in other groups (Table S1). The immunosuppressant regimen also differed between the groups. The proportion of TAC monotherapy was highest in the high exposure group (31.0%, 12.5%, 26.9%, and 68.0%, respectively) and that of TAC + mTORi showed a reverse trend with the degree of CET (22.7%, 81.2%, 48.4%, 2.0%, respectively). Oral steroids were maintained similarly in most of the patients in the four groups.

### HCC recurrence and overall death

**Figure 3:**
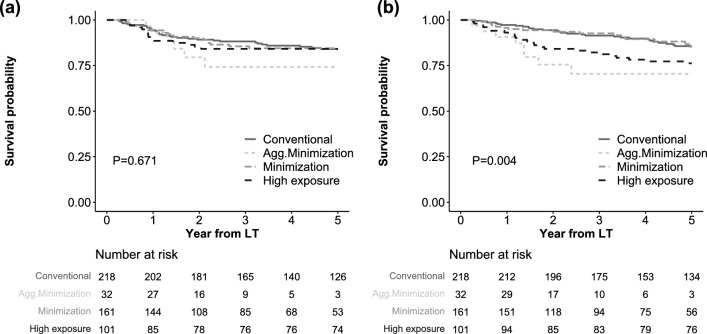
Kaplan–Meier analyses for survival outcomes. (**a**) HCC recurrence = free survival, (**b**) overall survival.

During a mean follow-up period of 43.2 ± 20.2 months, 74 (14.5%) patients experienced HCC recurrence, and 77 (15.0%) patients died. The median duration from LT to HCC recurrence was 13.4 (IQR 9.4–23.1) months (Figure S2). Using the Kaplan–Meier analysis, the 5-year recurrence-free survival was not significantly different between the four CET groups (84.0%, 74.2%, 84.5%, and 84.2% for conventional, aggressive minimization, minimization, and high exposure groups, respectively, P = 0.671; Fig. 3a). In contrast, the 5-year overall survival was significantly different between groups, showing lower survival in the aggressive minimization and high exposure groups than in the other groups (85.6%, 70.5%, 85.2%, and 76.2%, respectively, P = 0.004, Fig. 3b). The distribution of cause of death is shown in Figure S3, without statistically significant differences.

In univariate and multivariate Cox analyses (Table 2), the CET group was not a significant risk factor for HCC recurrence (HR 1.13, P = 0.790 for aggressive minimization; HR 0.99, P = 0.973 for minimization; HR 0.94, P = 0.848 for high exposure vs. conventional). Other significant risk factors for HCC recurrence were age (HR 0.95, P = 0.004), BMI (HR 0.92, P = 0.033), pre-transplant MELD scores (HR 0.94, P = 0.017), log AFP (HR 1.18, P = 0.018), bridging treatment (HR 0.21, P = 0.001 for none; HR 2.38, P = 0.003 for systemic or radiotherapy versus locoregional treatment), viable tumor number (HR 1.07, P = 0.006), maximum tumor size (HR 1.13, P = 0.044), microvascular invasion (HR 2.14, P = 0.005), and acute rejection within 3 months (HR 2.10, P = 0.006).

**Table 2:** Results of multivariable Cox analyses for HCC recurrence and overall death.

Variables	HCC recurrence ^a^			Overall death		
	HR	95% CI	P	HR	95% CI	P
Group by CET 90 days						
Conventional	1.00			1.00		
Aggressive minimization	1.23	0.50–3.03	0.652	5.64	2.18–14.63	< 0.001
Minimization	0.97	0.53–1.76	0.918	1.13	0.59–2.17	0.719
High exposure	0.93	0.50–1.75	0.833	1.67	0.94–2.95	0.081
Age	0.96	0.93–1.00	0.041	1.05	1.01–1.09	0.008
BMI	0.92	0.85–1.00	0.038	0.91	0.85–0.98	0.014
Pre-transplant MELD	0.95	0.90–1.00	0.042			
Deceased vs. living donor				0.50	0.30–0.84	0.008
Log AFP	1.16	1.02–1.33	0.028	1.19	1.05–1.34	0.006
Bridging treatment						
Locoregional	1.00			1.00		
None	0.22	0.09–0.57	0.002	0.79	0.42–1.46	0.449
Systemic or radiotherapy	2.27	1.29–3.98	0.004	3.55	1.93–6.55	< 0.001
Viable tumor number	1.07	1.01–1.12	0.014	1.05	1.00–1.10	0.064
Maximum tumor size	1.14	1.01–1.28	0.033			
Microvascular invasion	2.26	1.31–3.88	0.003			
Satellite nodule	1.83	1.02–3.27	0.043	4.57	2.59–8.06	< 0.001
Use of mTOR inhibitor				0.26	0.12–0.57	0.001
Biopsy proven rejection within 3 months				2.55	1.12–5.84	0.026

Covariates with P < 0.10 in univariable models were included in multivariable models.

Only variables that were significant in the multivariable model were demonstrated except CET 90 days group. The full results are provided in the Supplementary Material (Tables S5, S6).

*AFP* alpha-feto protein, *CET* cumulative exposure to tacrolimus, *HCC* hepatocellular carcinoma, *MELD* model for end-stage liver disease, *PIVKA II* protein induced by vitamin K absence or antagonist-II.

^a^Multivariable analyses for HCC recurrence were performed, treating non-HCC death as a competing risk.

In contrast, aggressive minimization was a significant risk factor for overall death in multivariate analyses (HR 5.19, P = 0.001) when compared to the conventional group. Minimization did not affect overall death (HR 1.16, P = 0.640), and the high-exposure group showed marginal risk (HR 1.64, P = 0.091) compared to the conventional group. Other risk factors for death were BMI (HR 0.91, P = 0.012), deceased donor (HR 2.00, P = 0.008), log AFP (HR 1.18, P = 0.007), systemic or radiotherapy versus locoregional therapy (HR 3.71, P < 0.001), viable tumor number (HR 1.06, P = 0.006), satellite nodule (HR 4.52, P < 0.001), and use of mTORi within 3 months (HR 0.25, P < 0.001).

### Matched and subgroup analyses for HCC recurrence

**Figure 4:**
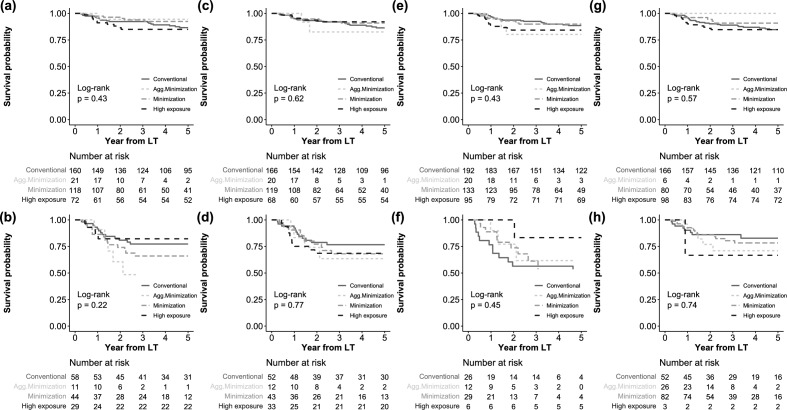
Subgroup analyses for HCC recurrence. (**a**) Within Milan, (**b**) above Milan, (**c**) French risk score ≤ 2, (**d**) French risk score > 2, (**e**) without systemic- or radiotherapy, (**f**) with systemic- or radiotherapy, (**g**) without mTORi, and (**h**) with mTORi.

We performed matched analyses for HCC recurrence between the conventional CET group and the other three CET groups. Baseline characteristics were well-balanced between matched groups (Tables S2–S4). The results also revealed no significant relationship between the CET groups and HCC recurrence (Fig. S4). We also performed subgroup analyses stratified by HCC recurrence risk, such as within/above Milan criteria, ≤ 2/ > 2 of French risk score, with/without systemic- or radiotherapy before LT, and with/without mTORi (Fig. 4). However, no significant difference was observed between the CET groups in these subgroups.

## Discussion

This study showed that TAC exposure during the first three months after LT did not affect HCC recurrence when analyzed based on CET calculated using all measured TAC trough levels. Overall death was significantly higher in the aggressive minimization group and marginally higher in the high-exposure group than in the conventional group. This study has some strengths; namely, the analysis of 512 LT populations only consisted of patients who had HCC before LT and who used TAC as the main immunosuppressant. Another outstanding aspect of this study was that the categorization of TAC exposure groups according to CET was calculated using all measured serum trough levels based on rigorous data collection. In contrast to previous observational studies, our results showed that TAC exposure during early period was not associated with HCC recurrence after LT.

Although TAC is a mainstream immunosuppressive agent in solid organ transplantation, it has considerable long-term adverse effects, such as renal impairment, hyperlipidemia, and increased malignancy^19^. Since the Symphony trial was introduced, low-dose TAC with a trough level of 3–7 ng/mL for 1 year after surgery has taken center stage in the kidney transplantation field^20^. However, subsequent studies revealed increased rejection rates from under-immunosuppression. This resulted in recent guidelines suggesting avoidance of TAC minimization unless a specific cause is present^21^. In the same guidelines for LT, recommendations for TAC levels were 6–10 ng/mL for the first month and 4–8 ng/mL thereafter, which was similar to the conventional dose category in the current study^21^. Low TAC was appropriate according to guidelines if there was concomitant use of MMF or mTOR inhibitors; however, the recommended therapeutic range was too wide to be effectively utilized in practice (4–12 ng/mL).

Unlike the well-established renal sparing benefit^22^, there is insufficient evidence for the effect of TAC on HCC recurrence after LT. More than a decade ago, Macro et al. reported that a high TAC exposure of > 10 ng/mL doubled HCC recurrence compared to conventional exposure^23^. However, the study had a small population of TAC users and possibly contained immortal time bias; more patients may have experienced an early recurrence of HCC in the high TAC group because it defined TAC exposure as the trapezoidal average of trough levels before HCC recurrence or death.

Rodriguez-Peralvarez et al. reported that high TAC doses (> 10 ng/mL) showed a 2.82-fold higher HCC recurrence than the lower TAC group via retrospective analysis with a population of 219 HCC patients^12^. However, the study defined TAC exposure as the mean trough level within 1 month after LT, which was too short to evaluate TAC exposure compared to the entire period of HCC recurrence, and was not enough to reflect intra-patient variability of TAC. In fact, TAC levels after 2 months were similar between the reduced- and high-exposure groups in that study. The same authors recently developed a novel CET method and reported that high TAC was related to a higher of de novo cancer after LT; however, it did not show robust results for HCC recurrence^16^. This study applied their relevant method for calculating CET and revealed that TAC exposure did not affect HCC recurrence after LT, even in the high-exposure group. When CET was analyzed as continuous variable, it was also not related with HCC recurrence (aHR 1.00, p = 0.760).

Regarding renal benefits, mTORi showed the most promising results with the TAC minimization protocol in LT patients^24,25^. For the prevention of HCC recurrence, prior studies showed the efficacy of mTORi^26–28^, although there were some discrepancies^29^. A recent meta-analysis showed that mTORi improved overall survival but did not reach statistical significance in HCC recurrence in LT recipients^30^. Furthermore, subsequent RCTs did not show a definite increase in recurrence free survival^13,14^. Although additional subgroup analyses of the SiLVER trial showed promising results for mTORi in patients with active HCC who used sirolimus for more than 3 months^31^, it was still unclear whether this effect resulted from mTORi or reduced TAC. In our study, mTORi was correlated only with overall death rather than HCC recurrence. When stratified by the use of mTORi, HCC death was similar whereas non-HCC death was lower in patients who used mTORi (Figure S5). Also, patients in Agg. minimization group showed different survival according to the use of mTORi, despite small number of population (Figure S6). We hypothesized that additional effect of mTORi such as renal protection or anti-viral effect could contribute this survival benefit, which could not be proven in this study setting. Further studies are needed to compare immunosuppression with mTORi, with reduced TAC and MMF, which is currently the most popular initial immunosuppressive regimen.

The aggressive minimization group seemed to be a high-risk population for HCC recurrence owing to the high tumor burden. Interpretation of outcomes other than overall death in that group was limited due to the small number (n = 32) of patients and the presence of a selection bias because the surgeon intended to use low TAC. However, multivariable and matched analyses showed no effect of aggressive minimization on HCC recurrence in this study. Furthermore, aggressive minimization was significantly associated with higher death compared to the Conventional group. Although the number of population and death in the aggressive minimization group was not enough to draw concrete evidence, one possible explanation was higher proportion of death from graft failure. Among patients who was dead, 37.5% (3 of 8) was from graft failure in aggressive minimization whereas 0 in conventional, 16.7% in minimization and 8.3% in High exposure group was death due to graft failure. This could be higher immunological damage in aggressive minimization group, however, no more evidence was found in this study. Further study using larger cohort is needed to validate our result about higher death under aggressive minimization of TAC early after LT.

The retrospective, single-center approach was a limitation of this study. Additionally, selection bias and small population size in the aggressive minimization group were other limitations that hindered the interpretation in that group. Lastly, living donor predominance, which resulted from the characteristics of the Korean LT circumstances, could limit the generalization of our results.

Despite these limitations, this study demonstrated that exposure to TAC during early period after did not affect the recurrence of HCC; rather, mortality increased with aggressive minimization of TAC. TAC minimization is not effective to prevent HCC recurrence but could result in higher mortality in LT recipients.

### Supplementary Information

Supplementary Information.

## Data Availability

The data that support the findings of this study are available from the corresponding author upon reasonable request.
