# Supplementary material for: Cumulative exposure to tacrolimus during early period after liver transplantation does not affect the recurrence of hepatocellular carcinoma
Source: Sci Rep. 2023 Nov 19;13:20236. doi: 10.1038/s41598-023-46803-8 (PMC10658176; doi:10.1038/s41598-023-46803-8)

**Supplement material. Detailed method for propensity matched analyses for HCC recurrence**

We performed matched analyses between Conventional group and other 3 CET groups, respectively. Propensity score was generated with covariates which were significant in multivariable Cox analyses for HCC recurrence, such as age, BMI, MELD, log AFP, type of bridging treatment, viable tumor number, maximum tumor size, microvascular invasion, and satellite nodule. Acute rejection within 3 months was not included because it was not baseline characteristics. Propensity score matching was performed using the nearest neighbor method with a caliper of 0.2 (1), with 1:3 manner for Agg.minimization vs. Conventional group due to small number of Agg.minimization group, while 1:1 manner for other two CET groups vs. Conventional group. We confirmed the absolute standardized mean differences of all covariates were under 0.2 as the upper limit of balance in the three matched cohorts.

1. Rubin DB. Using Propensity Scores to Help Design Observational Studies: Application to the Tobacco Litigation. Health Services and Outcomes Research Methodology. 2001;2(3):169-88.

**Figure S1. Comparison of immunosuppressant use**

**Table S1. Comparison of immunosuppressant use at 3 months**

| **Variables** | **Conventional**  **(n=218)** | **Agg.minimization**  **(n=32)** | **Minimization**  **(n=161)** | **High exposure**  **(n=101)** | **P** |
| --- | --- | --- | --- | --- | --- |
| Intra-patient variability of TAC trough level |  |  |  |  |  |
| SD | 3.0 (2.2-3.7) | 1.7 (1.1-2.1) | 2.0 (1.5-2.7) | 3.8 (3.1-4.9) | <0.001 |
| Variance | 8.9 (4.8-14.0) | 2.7 (1.2-4.4) | 4.0 (2.4-7.4) | 14.7 (9.6-23.7) | <0.001 |
| Coefficient of variation | 37.4 (29.0-45.5) | 48.9 (34.9-66.1) | 36.8 (27.9-47.1) | 33.8 (27.9-42.5) | 0.001 |
| Immunosuppressants regimen |  |  |  |  |  |
| TAC | 67 (31.0) | 4 (12.5) | 43 (26.9) | 68 (68.0) | <0.001 |
| TAC+MMF | 100 (46.3) | 2 (6.2) | 39 (24.4) | 30 (30.0) |  |
| TAC+mTORi | 49 (22.7) | 26 (81.2) | 78 (48.4) | 2 (2.0) |  |
| Oral steroid | 215 (98.6) | 31 (96.9) | 160 (99.4) | 98 (97.0) | 0.426 |

**Figure S2. Density plot for month from LT to HCC recurrence**

**
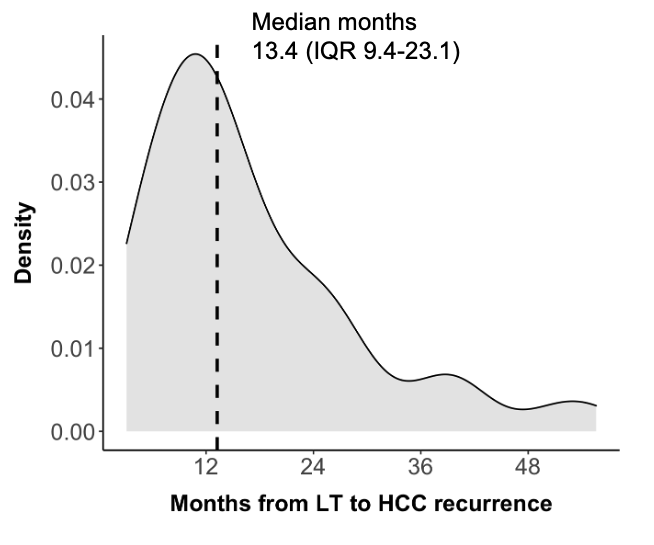
**

**Figure S3. Cause of death in each CET group.**

**
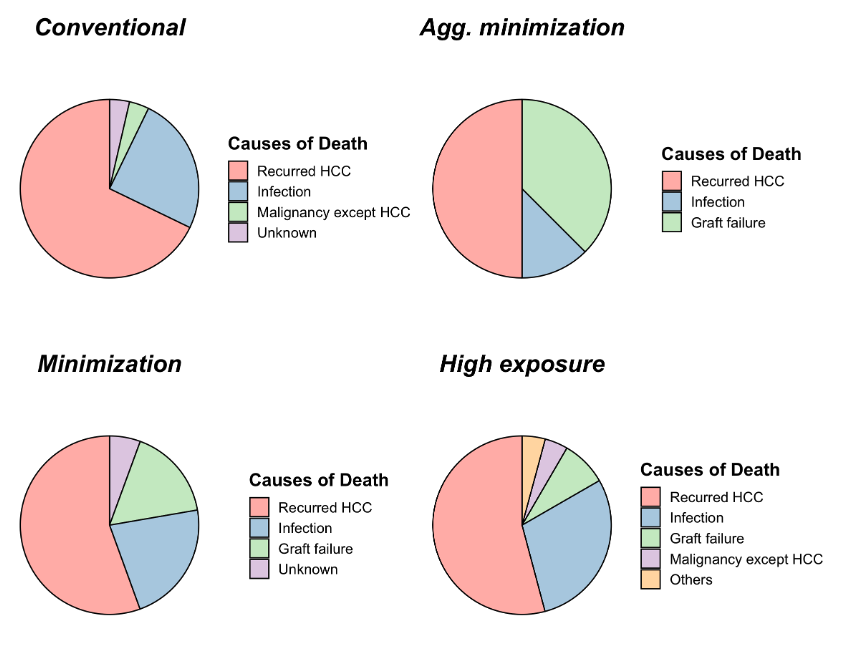
**

**Figure S4. Matched analyses for HCC recurrence between Conventional group vs. other CET groups**

**Table S2. Baseline characteristics in matched groups; conventional vs. Agg. minimization**

| **Variables** | **Conventional**  **(n=70)** | **Agg.minimization**  **(n=29)** | **P** |
| --- | --- | --- | --- |
| Age, year | 58.3 ± 7.5 | 58.5 ± 8.0 | 0.885 |
| Sex, female | 61 (87.1) | 20 (69.0) | 0.065 |
| BMI, kg/m^2^ | 23.9 (23.1-25.6) | 23.9 (22.3-26.0) | 0.370 |
| Underlying for HCC |  |  | 0.472 |
| Hepatitis B | 53 (75.7) | 20 (69.0) |  |
| Hepatitis C | 7 (10.0) | 2 (6.9) |  |
| Non-B, Non C | 10 (14.3) | 7 (24.1) |  |
| ABO incompatibility | 17 (24.3) | 5 (17.2) | 0.616 |
| Hypertension | 27 (38.6) | 8 (27.6) | 0.418 |
| Diabetes mellitus | 26 (37.1) | 15 (51.7) | 0.264 |
| Pre-transplant MELD | 11.0 (8.0-15.0) | 11.0 (8.0-19.0) | 0.502 |
| Donor type |  |  | 0.302 |
| Living | 52 (74.3) | 25 (86.2) |  |
| Deceased | 25 (35.7) | 4 (13.8) |  |
| Donor age, year | 35.0 (28.0-41.0) | 38.0 (29.0-46.0) | 0.489 |
| Donor sex, female | 45 (64.3) | 17 (58.6) | 0.763 |
| Graft steatosis >10% | 5.0 (0.0-5.0) | 5.0 (0.0-5.0) | 0.740 |
| AFP, ng/mL | 2.0 (1.1-3.4) | 1.9 (1.1-3.2) | 0.718 |
| PIVKA II, mAU/mL | 3.7 (2.9-4.9) | 4.9 (3.5-6.0) | 0.016 |
| Salvage LT | 9 (12.9) | 1 (3.4) | 0.295 |
| Bridging treatment |  |  | 0.812 |
| None | 17 (24.3) | 8 (27.6) |  |
| Locoregional | 29 (41.4) | 10 (34.5) |  |
| Systemic or radiotherapy | 24 (34.3) | 11 (37.9) |  |
| Total necrosis | 12 (17.1) | 5 (17.2) | 1.000 |
| Viable tumor number | 2.0 (1.0-3.0) | 1.0 (1.0-4.0) | 0.617 |
| Maximum viable tumor size | 1.8 (1.1-3.0) | 1.5 (1.1-3.0) | 0.823 |
| Sum of viable tumor size | 3.2 (1.2-5.5) | 1.9 (1.1-6.2) | 0.515 |
| Microvascular invasion | 18 (25.7) | 6 (20.7) | 0.785 |
| Poor differentiation | 18 (25.7) | 13 (44.8) | 0.103 |
| Satellite nodule | 9 (12.9) | 2 (6.9) | 0.612 |
| Above Milan criteria | 28 (40.0) | 10 (34.5) | 0.774 |
| French risk score >2 | 24 (34.3) | 10 (34.5) | 1.000 |

**Table S3. Baseline characteristics in matched groups; conventional vs. minimization**

| **Variables** | **Conventional**  **(n=147)** | **Minimization**  **(n=147)** | **P** |
| --- | --- | --- | --- |
| Age, year | 58.0 (52.0-62.0) | 57.0 (52.5-62.0) | 0.858 |
| Sex, female | 122 (83.0) | 114 (77.6) | 0.305 |
| BMI, kg/m^2^ | 24.1 (22.7-26.0) | 23.9 (22.2-26.3) | 0.554 |
| Underlying for HCC |  |  | 0.856 |
| Hepatitis B | 114 (77.6) | 110 (74.8) |  |
| Hepatitis C | 12 (8.2) | 13 (8.8) |  |
| Non-B, Non C | 21 (14.3) | 24 (16.3) |  |
| ABO incompatibility | 33 (22.4) | 27 (18.4) | 0.469 |
| Hypertension | 39 (26.5) | 35 (23.8) | 0.687 |
| Diabetes mellitus | 44 (29.9) | 50 (34.0) | 0.532 |
| Pre-transplant MELD | 10.0 (8.0-14.0) | 12.0 (8.0-15.0) | 0.145 |
| Donor type |  |  | 0.218 |
| Living | 117 (79.6) | 126 (85.7) |  |
| Deceased | 30 (20.4) | 21 (14.3) |  |
| Donor age, year | 31.0 (25.0-42.0) | 35.0 (26.0-42.0) | 0.305 |
| Donor sex, female | 95 (64.6) | 88 (59.9) | 0.470 |
| Graft steatosis >10% | 5.0 (0.0-5.0) | 5.0 (0.0-5.0) | 0.498 |
| AFP, ng/mL | 1.8 (1.2-2.9) | 1.7 (1.1-2.9) | 0.434 |
| PIVKA II, mAU/mL | 3.5 (3.0-4.4) | 3.6 (3.0-4.5) | 0.473 |
| Salvage LT | 22 (15.0) | 15 (10.2) | 0.291 |
| Bridging treatment |  |  | 0.971 |
| None | 37 (25.2) | 38 (25.9) |  |
| Locoregional | 89 (60.5) | 87 (59.2) |  |
| Systemic or radiotherapy | 21 (14.3) | 22 (15.0) |  |
| Total necrosis | 27 (18.4) | 30 (20.4) | 0.768 |
| Viable tumor number | 2.0 (1.0-3.0) | 1.0 (1.0-3.0) | 0.090 |
| Maximum viable tumor size | 1.7 (1.0-2.5) | 1.6 (0.9-2.5) | 0.759 |
| Sum of viable tumor size | 2.7 (1.1-5.0) | 2.2 (0.9-4.4) | 0.261 |
| Microvascular invasion | 33 (22.4) | 30 (20.4) | 0.776 |
| Poor differentiation | 36 (24.5) | 53 (36.1) | 0.042 |
| Satellite nodule | 14 (9.5) | 10 (6.8) | 0.523 |
| Above Milan criteria | 46 (31.3) | 35 (23.8) | 0.192 |
| French risk score >2 | 39 (26.5) | 35 (23.8) | 0.687 |

**Table S4. Baseline characteristics in matched groups; conventional vs. high exposure**

| **Variables** | **Conventional**  **(n=93)** | **High exposure**  **(n=93)** | **P** |
| --- | --- | --- | --- |
| Age, year | 54.2 ± 7.2 | 53.9 ± 6.7 | 0.744 |
| Sex, female | 78 (83.9) | 79 (84.9) | 1.000 |
| BMI, kg/m^2^ | 24.4 ± 2.9 | 24.3 ± 3.0 | 0.777 |
| Underlying for HCC |  |  | 0.260 |
| Hepatitis B | 75 (80.6) | 83 (89.2) |  |
| Hepatitis C | 7 (7.5) | 4 (4.3) |  |
| Non-B, Non C | 11 (11.8) | 6 (6.5) |  |
| ABO incompatibility | 21 (22.6) | 5 (5.4) | 0.002 |
| Hypertension | 24 (25.8) | 20 (21.5) | 0.605 |
| Diabetes mellitus | 26 (28.0) | 21 (22.6) | 0.500 |
| Pre-transplant MELD | 9.0 (7.0-12.0) | 9.0 (7.0-12.0) | 0.633 |
| Donor type |  |  | 0.388 |
| Living | 74 (79.6) | 68 (73.1) |  |
| Deceased | 19 (20.4) | 25 (26.9) |  |
| Donor age, year | 32.0 (25.0-43.0) | 34.0 (25.0-46.0) | 0.692 |
| Donor sex, female | 55 (59.1) | 68 (73.1) | 0.063 |
| Graft steatosis >10% | 5.0 (0.0-5.0) | 5.0 (0.0-5.0) | 0.793 |
| AFP, ng/mL | 1.9 (1.4-3.2) | 2.2 (1.4-3.4) | 0.504 |
| PIVKA II, mAU/mL | 3.5 (2.9-4.3) | 3.3 (2.8-4.1) | 0.120 |
| Salvage LT | 18 (19.4) | 16 (17.2) | 0.850 |
| Bridging treatment |  |  | 0.914 |
| None | 20 (21.5) | 22 (23.7) |  |
| Locoregional | 66 (71.0) | 65 (69.9) |  |
| Systemic or radiotherapy | 7 (7.5) | 6 (6.5) |  |
| Total necrosis | 14 (15.1) | 13 (14.0) | 1.000 |
| Viable tumor number | 2.0 (1.0-4.0) | 1.0 (1.0-3.0) | 0.295 |
| Maximum viable tumor size | 1.7 (1.0-2.5) | 1.8 (1.0-3.0) | 0.383 |
| Sum of viable tumor size | 3.5 (1.1-4.7) | 3.0 (1.1-5.7) | 0.995 |
| Microvascular invasion | 21 (22.6) | 23 (24.7) | 0.863 |
| Poor differentiation | 25 (26.9) | 20 (21.5) | 0.493 |
| Satellite nodule | 11 (11.8) | 12 (12.9) | 1.000 |
| Above Milan criteria | 36 (38.7) | 26 (28.0) | 0.162 |
| French risk score >2 | 32 (34.4) | 27 (29.0) | 0.529 |

**Table S5. Full results of multivariable Cox analyses for HCC recurrence**


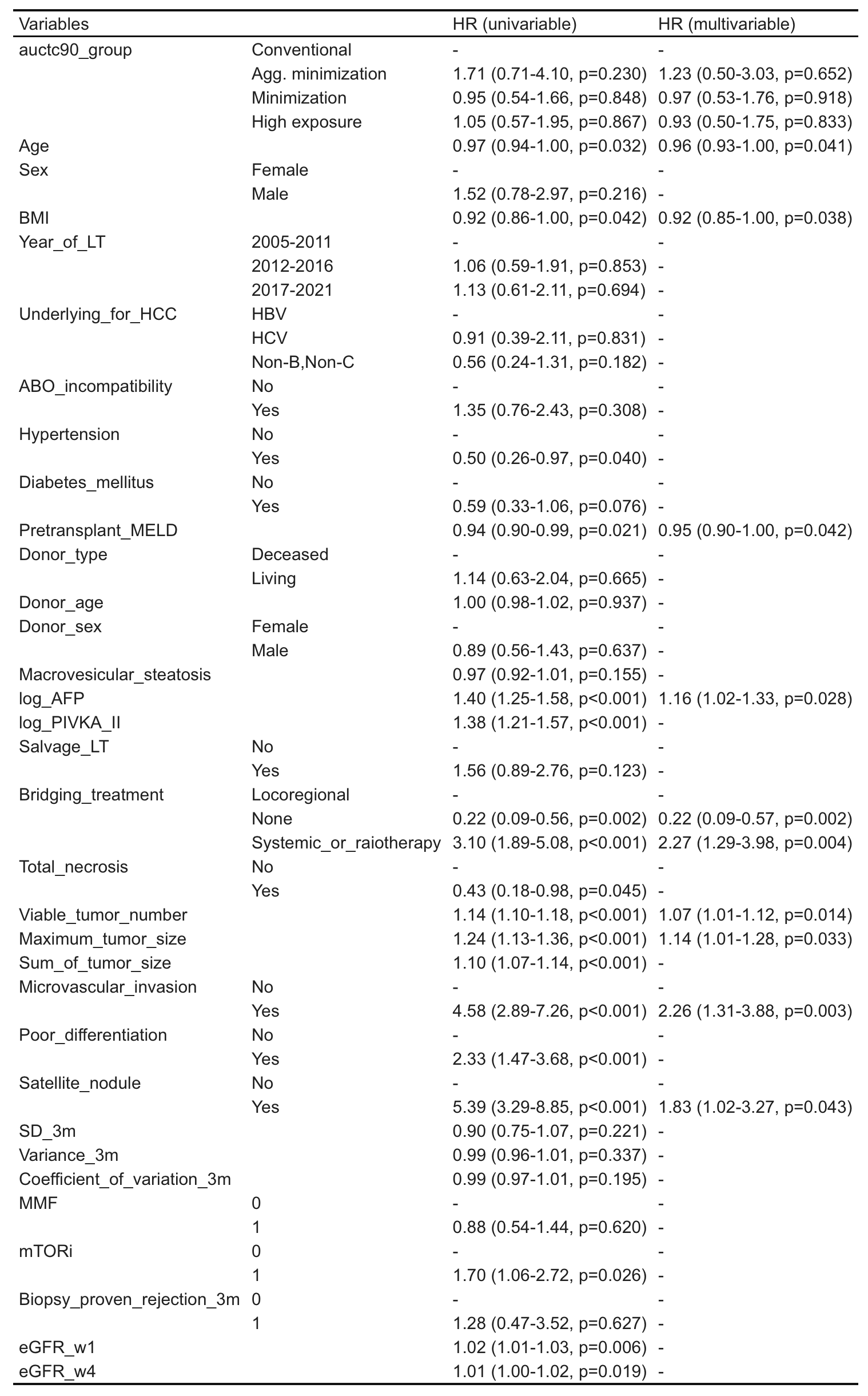


**Table S6. Full results of multivariable Cox analyses for overall death**


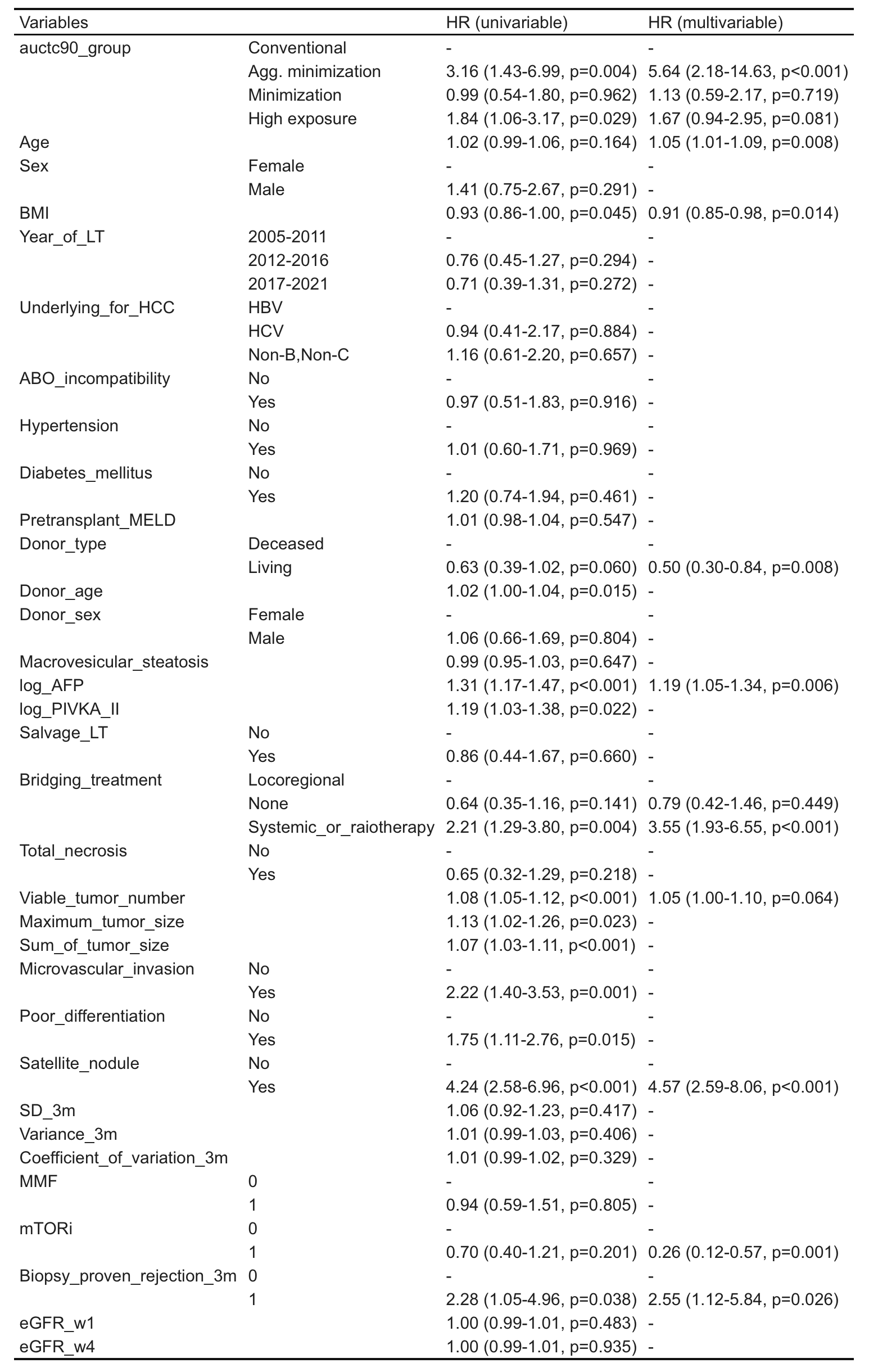


**Figure S5. Comparison of HCC death and non-HCC death between according to the use of mTORi**


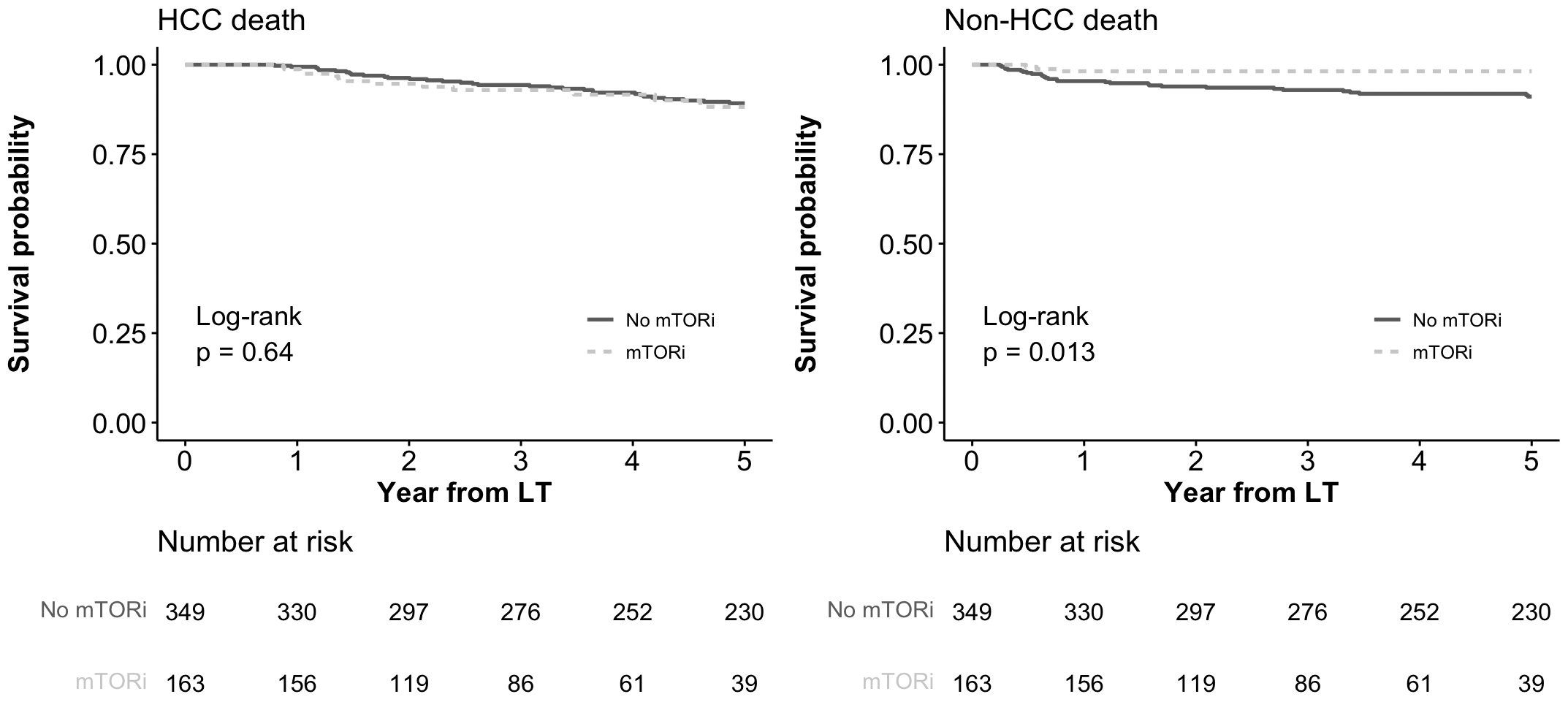


**Figure S6. Patient survival of Agg.minimization group according to the use of mTORi**


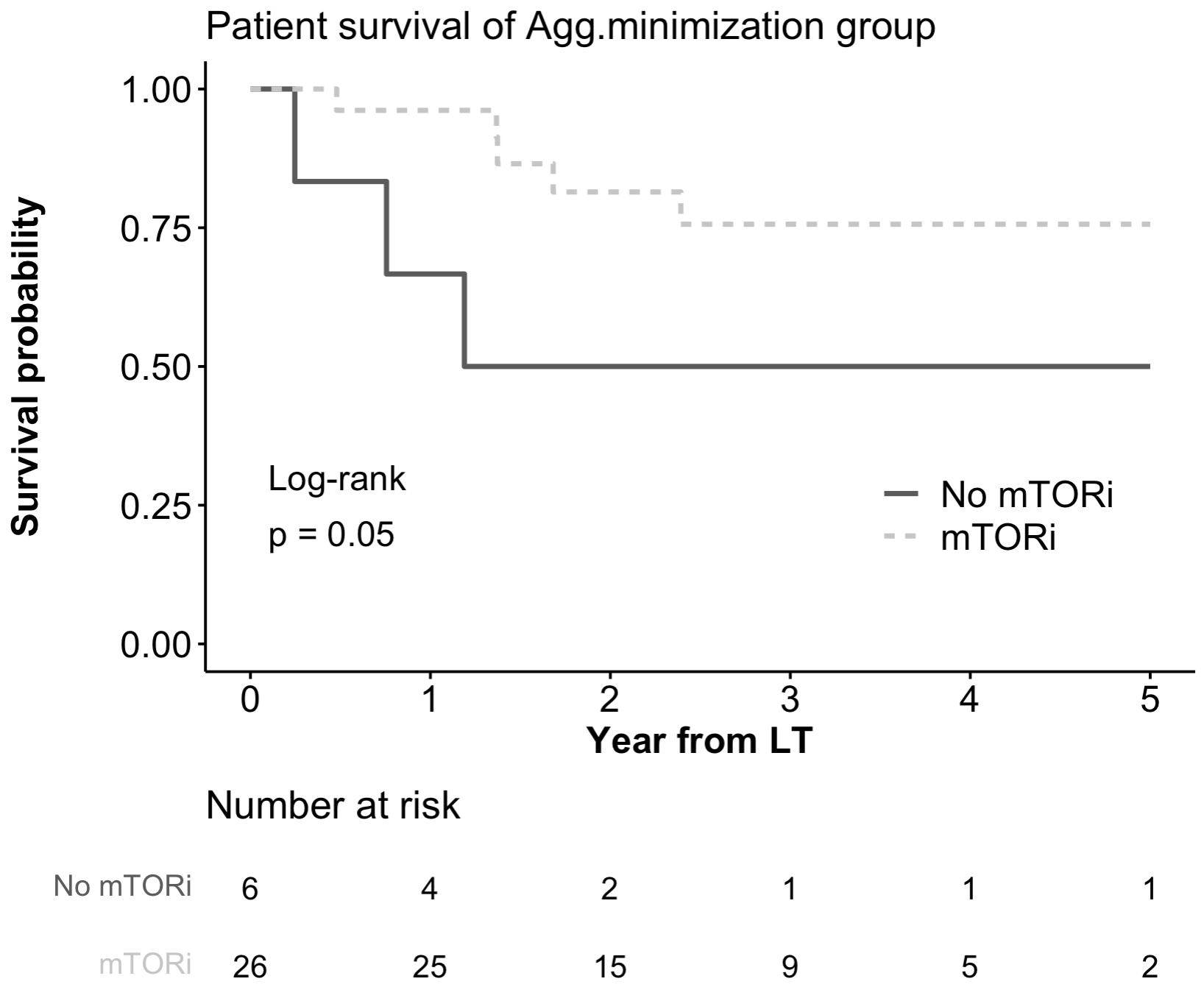

Supplement: Supplementary file 1 — Supplementary Information. [file 41598_2023_46803_MOESM1_ESM.docx]
